# PLA/PCL Polymer Material for Food Packaging with Enhanced Antibacterial Properties

**DOI:** 10.3390/polym17091134

**Published:** 2025-04-22

**Authors:** Krzysztof Moraczewski, Magdalena Stepczyńska, Aneta Raszkowska-Kaczor, Lauren Szymańska, Piotr Rytlewski

**Affiliations:** 1 Faculty of Materials Engineering, Kazimierz Wielki University, Chodkiewicza 30 St., 85-064 Bydgoszcz, Poland; m.stepczynska@ukw.edu.pl (M.S.); prytlewski@ukw.edu.pl (P.R.); 2 Łukasiewicz Research Network–Institute of Polymer Materials, M. Skłodowska-Curie St., 87-100 Toruń, Poland; aneta.kaczor@impib.lukasiewicz.gov.pl (A.R.-K.);

**Keywords:** polylactide, polycaprolactone, tannic acid, active food packaging, antibacterial

## Abstract

Active food packaging is a significant trend in recent years in the food industry. This paper presents the results of studies on selected properties of a mixture of polylactide and polycaprolactone containing 1 or 5 wt.% of tannic acid. The function of tannic acid was to improve the miscibility of the polymers used and to give the obtained composition antibacterial properties. Studies were carried out on color and transparency, microscopic analysis, water vapor permeability, mass flow rate, static tensile properties, impact strength, dynamic mechanical analysis, thermogravimetry and differential scanning calorimetry. The obtained results did not confirm the compatibilizing effect of tannic acid, because the obtained mechanical properties were slightly worse than those of materials without the addition of this compound. However, the obtained mixture was characterized as having biocidal properties against two strains of *Escherichia coli* (ATCC 8739) and *Staphylococcus aureus* (ATCC 6538P). Antibacterial properties together with acceptable processing, mechanical and thermal properties indicate that the presented polymer material may be a potential material for the production of active food packaging.

## 1. Introduction

In today’s world, where environmental care is a lifestyle rather than a trend, the need to protect nature is essential. We are entering an era of ecological food packaging, which, due to its short use, quickly becomes waste. Packaging production is a major contributor to this issue. In response, companies are adopting solutions to minimize environmental impact. As a result, ecologists, policymakers, and environmental advocates are promoting the use of eco-friendly packaging made from biodegradable and compostable polymers.

Biodegradable packaging is an important part of a wide range of packaging. Biodegradable polymers can be used to pack food products and more. These materials meet all quality requirements, and have the appropriate approvals and certificates. Many biodegradable polymers have a high barrier to oxygen and water vapor, and are also weldable, mechanically durable, and can be used on typical packaging machines.

Biodegradable polymers used in packaging production can be classified into the following groups [1]:

- polymers isolated from plant and animal biomass, including polysaccharides, lipids and proteins;
- polymers synthesized by microorganisms or genetically modified plants, polyhydroxyalkanoates (PHA);
- polymers synthesized chemically from monomers obtained in the fermentation process of biomass, e.g., polylactide (PLA), polymers obtained from petrochemical raw materials: polycaprolactones (PCL), polyesteramides (PEA), aliphatic copolyesters (PBSA).

Of the polymers mentioned above, polylactic acid (PLA) is most widely used in the production of food packaging [2,3,4,5]. The properties of this polymer are particularly useful in the production of packaging. PLA can be used for various applications; for example, for packaging fruits and vegetables. Thermoformed trays and containers, as well as shopping bags and even labels are made from this polymer. It is suitable for processing on conventional thermoforming and extrusion equipment. PLA is a polymer that is compostable under industrial conditions and packaging made from it has certificates in accordance with the EN13432:2000 standard issued by DIN CERTCO and is authorized to apply the composability mark. Additionally, to compensate for certain disadvantages of PLA (low impact strength), very often in the production of food packaging, mixtures of PLA with other polymers or compounds are used, including polycaprolactone (PCL), polyhydroxybutyrate (PHB), PBAT, chitosan, cellulose and its derivatives and starch [6,7,8].

An important trend in recent years in the production of food packaging is so-called active packaging. Active packaging affects the packaged product or changes the conditions inside the package, extending the durability of the product and maintaining its high quality [9]. The impact of such packaging is the result of additional substances contained in it, such as antimicrobial compounds, fragrances, dyes, vitamins or antioxidants.

Active materials containing antimicrobial compounds are of particular importance in the production of food packaging. The development and implementation of antibacterial packaging is a promising way to actively control the development of bacteria and fungi that lead to food spoilage. The use of antibacterial materials in food packaging extends the shelf life and slows down the development of microorganisms, thus ensuring better quality and safety of meat, vegetables, fruits and dairy products [10].

Packaging with antimicrobial properties is obtained by introducing biologically active substances into the polymer matrix by their adsorption or immobilization using covalent or ionic bonds [11]. A wide range of antimicrobial agents have been described in the literature, such as metallic nanoparticles (Ag, Cu, bimetallic, etc.) [12,13], oxide nanoparticles (ZnO, TiO_2_, CuO etc.) [14,15], clay nanoparticles (cloisite, montmorillonite, bentonite) [16,17], natural extracts (essential oils or hydrophilic extracts) [18,19], natural antimicrobials (nisin, pediocin, natural antibiotics, etc.) [20,21], biopolymers (chitosan) [22,23], enzymes (lysozyme, peroxidase) [24,25] and synthetic antimicrobials (including synthetic antibiotics) [26].

The aim of the conducted research was to determine the possibility of using tannic acid as an antimicrobial agent added to the PLA/PCL polymer mixture, thus creating an active material that can be used in the production of food packaging. An additional effect of using tannic acid was to be a compatibilizing effect, increasing the miscibility of these polymers and improving selected properties of the obtained mixture. Tannic acid is a well-known organic compound that has received considerable attention in many fields. Tannic acid is a type of high molecular-weight polyphenol that can be found in different parts of plants and trees, such as seeds, fruits, roots, and bark.

Polyphenols are a very broad group of chemical compounds, which include, among others, phenolic acids, flavonoids, tannins, and ellagitannins. These compounds are already used in the processes of obtaining active food packaging [27,28,29]; however, there is no information in the literature about the use of pure tannic acid in the production of active food packaging, especially those made from a PLA/PCL mixture. There is some information about the use of this compound in the production of packaging, however, the function of tannic acid in such cases is cross-linking or antioxidant action, and those presented results do not include testing the antibacterial effect [30,31]. The presented research results therefore have elements of scientific novelty, and the results obtained will answer the question of the possibility of using tannic acid as an active antibacterial ingredient of a film made of a mixture of polylactide and polycaprolactone

## 2. Materials and Methods

### 2.1. Materials

The following materials were used in the research. Polymer materials were polylactide (PLA) Nature Works 2003D (Cargill Dow LLC, Minneapolis, MN, USA) and polycaprolactone (PCL) CAPA 6800 (Perstorp, Warrington, UK). The PLA 2003D polymer has a tensile strength of 60.0 MPa, a elongation at break of 6.0%, a glass transition temperature of 55–60 °C, a melting temperature of 145–160 °C, a density of 1.24 g/cm^3^, and a melt flow rate of 6 g/10 min (210 °C/2.16 kg). The PCL CAPA 6800 has a tensile strength of 14.0 MPa, an elongation at break of 800.0%, a glass transition temperature of −62–−58 °C, a melting temperature of 58–60 °C, a density of 1.10 g/cm^3^, and a melt flow rate of 4 g/10 min (160 °C/2.16 kg). The addition to the polymer matrix was tannic acid C_76_H_52_O_46_ (Sigma-Aldrich, St. Louis, MO, USA; Burlington, MA, USA) with a molecular weight of 1701.20 g/mol and density of 2.12 g/cm^3^.

### 2.2. Sample Preparation

The tested polymer materials were produced using two processing methods: extrusion and injection molding. The extrusion process produced granulate of new polymer materials. Before the extrusion process, the individual components of the obtained polymer material were mixed in appropriate proportions. The matrix of the tested materials was a mixture of PLA and PCL. The main component of the new polymer material was PLA in the amount of 85 or 70 wt.%. The second polymer component of the tested materials was PCL in the amount of 15 or 30 wt.%. The content of tannic acid in the tested materials was 1 or 5 wt.%. The concentration of tannic acid was related to the mass of the PLA/PCL mixture.

Extrusion was carried out using a single-screw extruder type W25-30D (Metalchem, Warsaw, Poland). The temperatures of the individual extruder zones and the die-head were 175, 185, 195, 195 °C, respectively. Intensive mixing screws were used, additionally containing retracting and kneading segments. After leaving the die-head, the material was cooled with an air stream and then granulated.

The granulate was then used to produce test samples using the injection molding method. Injection molding was carried out using a screw injection molding machine TRX 80 Eco series (Tederic, Hangzhou, China) to manufacture standardized type 1A dumbbells. The sample dimensions (standardized type 1A dumbbells) were as follows: thickness 4.0 ± 0.2 mm, width of the gage section 10.0 ± 0.2 mm, length of the gage section 80.0 ± 0.2 mm and a total length over 150 mm. The temperatures of the subsequent zones of the injection molding machine were 170, 165 and 165 °C. The nozzle temperature was 165 °C. The mold was thermostated at 35 °C. The applied injection and holding pressures were 60 and 58 bar, respectively. The applied cooling time of the samples in the mold was 60 s.

As a result, research samples were created, the designation and composition of which are presented in Table 1.

### 2.3. Color and Transparency

Color measurements were performed using a BYK colorimeter (BYK-Gardner GmbH, Geretsried, Germany), designed for measuring the color and gloss of polymer materials. The color measurement was quantified using the color space standardized by the CIELab system based on three components: L, a and b. The “L” component indicates the lightness of the sample, the “a” component indicates the color from green to red, and the “b” component indicates the color from blue to yellow. The difference between two colors in the CIELab space (ΔE) was determined from Formula (1) and is the ordinary Euclidean distance between two points in three-dimensional space:

(1) $$∆E=\sqrt{{∆L}^{2}+{∆a}^{2}+{∆b}^{2}}$$

where ΔL is the difference in L between two samples while, similarly, Δa and Δb are the differences in the a and b coordinates, respectively.

For samples without tannic acid, the ΔL value was the difference between the PLA sample and the PLA/PCL mixture samples. Whereas for samples containing tannic acid, the ΔL value was the difference between the corresponding PLA/PCL mixture sample and the PLA/PCL mixture sample with tannic acid.

The transparency of the obtained materials was determined in two ways. In the first method was determined indirectly by assessing the degree of visibility of an inscription printed on a sheet of paper covered with a 320 μm thick sheet. The sheet was obtained by pressing the test sample using a hydraulic press. In the second method, the Evolution 220 (Thermo Scientific, Waltham, MA, USA) spectrometer with a beam focusing system enabling the examination of solid materials was used. The measurement was performed in the spectral range from 190 to 1100 nm. The level of material transparency was defined as the transmittance value of UV radiation at a wavelength of 550 nm [32].

### 2.4. Microscopic Analysis

Scanning electron microscopy (SEM) analyses were performed using a Phenom XL (Thermo Scientific, USA) microscope equipped. The samples were mounted on holders using conductive carbon tape to ensure charge dissipation. Before imaging, the sample surfaces were coated with a gold layer using a low-vacuum coater MCM100P (SEC, Seoul, Republic of Korea) for 2 min, achieving a conductive layer sufficient for charge dissipation during SEM observations. SEM imaging was performed at magnifications ranging from 300× to 5000×, with an accelerating voltage of 10–15 kV, using the BSD Full (Back Scatter Detector) mode in high vacuum (0.1 Pa). The field of view (FOV) ranged from 51.6 µm to 955 µm, with a working distance of 7.6–8.3 mm.

### 2.5. Water Vapor Permeability

The measurement of water vapor permeability was based on the precise determination of the mass loss of water that evaporated from the interior of a specialized test chamber through the examined sample. The penetration of water vapor occurs due to an increase in its partial pressure resulting from the rise in water temperature inside the test chamber. The assessment of water vapor permeability was conducted using a Radwag MAX 60/NH (Radwag, Radom, Poland) moisture analyzer. The study was conducted on 320 μm thick sheet obtained by pressing the individual test sample using a hydraulic press. The final result for a given material was the arithmetic mean of three measurements.

The applied research methodology assumes that the constant mass loss of water occurs 60 min after the commencement of the test. The initial phase of the analysis cannot be included in the measurements, as the conditions of the analysis are still unstable. During this period, the drying chamber stabilizes, and consequently, the conditions for conducting the analysis also stabilize. After this time, the permeability reaches a constant level—Δm/dΔt = const. Therefore, for the calculations, mass measurements taken at the 60th and 120th minute of the test were used. The water vapor permeability value was calculated using the following formula:

(2) $$P=\frac{M_{1}-M_{2}}{t\times P_{p}}\;\left[\frac{mg}{{cm}^{2}\times h}\right]$$

where *P*—water vapor permeability; *M*_1_—mass of distilled water recorded at 60 min of the test; *M*_2_—final mass of distilled water recorded at 120 min of the test; *t*—test time; *P_p_*—evaporation surface area (19.625 cm^2^).

### 2.6. Melt Flow Rate

The melt flow rate (MFR) test was performed using a capillary plastometer type MP600 (Tinius Olsen, Horsham, PA, USA). The melt flow rate was determined at 190 °C with a piston load of 2.16 kg. The MFR values of individual materials were determined based on the results of measurements of 12 samples. The arithmetic mean of 10 measurements was adopted as the value characterizing each of the tested materials (2 extreme measurements were rejected).

### 2.7. Mechanical Properties

Static tensile tests were performed on an Instron 3367 (Instron, Norwood, MA, USA) testing machine at a strain rate of 50 mm/min. Tensile tests were performed on standardized samples in the form of dumbbells. The values of tensile strength (σ_M_) and relative elongation (ε_B_) at break of individual materials were determined based on the results of measurements of 12 samples. The arithmetic mean of 10 measurements was adopted as the value characterizing each of the tested materials (2 extreme measurements were rejected).

Charpy impact tests were carried out using an XJ 5Z impact hammer (Liangong, Shandong, China) with a 2 J impact energy and a fall speed of 2.9 m/s. The tests were carried out on samples in the form of unnotched bars with dimensions of 80 × 10 × 4 mm cut from the gage of injection-molded tensile strength test samples. The unnotched impact strength value (u_a_) was determined as part of the test. The arithmetic mean of 10 measurements was adopted as the value characterizing each of the tested materials (2 extreme measurements were rejected).

### 2.8. Thermomechanical Properties

Thermomechanical tests were performed using a DMA Q800 dynamic mechanical analyzer (TA Instruments, New Castle, DE, USA). DMA tests were performed in an air atmosphere, in the temperature range of 30 to 150 °C with a temperature change rate of 3 °C/min. The strain was 15 μm and the strain frequency was 1 Hz. The samples were cuboids with dimensions of 60 × 10 × 4 mm cut from the gage of injection-molded tensile strength test samples. For each sample, the change in the storage modulus (E’), the damping coefficient (K) and the thermomechanical glass transition temperature (T_gDMA_) were determined. The E’ values were determined at temperatures of 30 °C (room temperature), 50 °C (before glass transition), 70 °C (after glass transition), 100 °C (before the cold crystallization process) and 130 °C (after the cold crystallization process).

### 2.9. Thermal Properties

Thermogravimetric analysis (TGA) tests were carried out using a TG Q500 thermobalance (TA Instruments, New Castle, DE, USA) in a nitrogen atmosphere in the temperature range of around 25 to 700 °C. The applied temperature change rate was 10 °C/min. The tested samples had a mass of 14.5 to 15.5 mg. Based on the thermogravimetric curve, a temperature value corresponding to the loss of 5, 10, 50 and 95% of the initial sample mass, as well as residue (R) was determined. The value of 5% mass loss was adopted as a parameter defining the thermal resistance of the material (T_d_). Full results of TGA are presented in Table S1 in the Supplementary Files.

Differential scanning calorimetry (DSC) studies were performed using a DSC Q200 instrument (TA Instruments, USA) in a nitrogen atmosphere in the temperature range of 0 to 200 °C. The applied temperature change rate was 10 °C/min. Samples weighing from 8.4 to 8.8 mg were subjected to 3 temperature change cycles (heating, cooling and a second heating cycle). The first heating cycle was to remove the thermal history of the materials. Based on the cooling and second heating curves, the glass transition temperature (T_g_), crystallization temperature (T_c_), change in crystallization enthalpy (ΔH_c_), cold crystallization temperature (T_cc_), change in cold crystallization enthalpy (ΔH_cc_), melting temperature (T_m_) and change in melting enthalpy (ΔH_m_) of the individual components of the polymer mixture and the degree of crystallinity (X_c_) were determined. The X_c_ values of the individual polymer phases of the mixture were calculated based on Formula (3). However, the results of X_c_ calculations are not presented in the article.

(3) $$X_{c}=\left(\frac{{∆H}_{m}}{{∆H}_{m100\%}\cdot \left(1-x\right)}\right)\cdot 100\%$$

where ΔH_m100%_—value of the enthalpy change of 100% crystalline sample: PLA 93 J/g [33], PCL 139.5 J/g [34], x—amount of a given polymer in the mixture.

### 2.10. Biocidal Properties

The evaluation of the bactericidal properties of the prepared samples was performed according to the standard ISO 22196 [35]. Two strains of model bacteria, i.e., *Escherichia coli* (ATCC 8739) and *Staphylococcus aureus* (ATCC 6538P), were used in the research. Both bacterial strains were maintained in sterile Tryptone Soya Agar (TSA) broth for 24 h at 37 °C. After this time, 1 mL of the obtained cultures were collected into sterile Eppendorf tubes and centrifuged for 10 min at 10,000 rpm. Then, the supernatant was removed, and the bacterial pellet was suspended in a sterile saline solution. Subsequently, the resulting suspension was adjusted to an optical density of 1.0, which corresponds to a cell count of 3.0 × 10^8^ in 1 mL. From the suspension obtained in this way, 50 µL was transferred to the surfaces of the tested plates. The whole plate was placed in a humid chamber for a contact time of 24 h at 20 °C. After this time, bacterial cells were recovered from the surface and suspended in a solution containing soybean casein digest broth with lecithin as a neutralizer. Then, the number of cells which were living and able to grow was investigated by inoculation on Plate Count Agar in triplicate. The plates were incubated at 37 °C for 24 h.

According to the standard, the bactericidal effect of the investigated samples was calculated using Formula (4). The reduction (R) of the number of living cells of the tested bacteria by at least two orders of magnitude was considered as biocidal effect.

R = l_0_ − l_W_ (4)

where l_0_—the logarithm of the number of cells in the control sample; l_W_—the logarithm of the number of cells in the test sample.

The microscopic images were performed in parallel with the bactericidal test. The epi-fluorescence images were created for the stained bacterial cells by using the LIVE/DEAD test to differentiate between the living and dead cells. After being in a humid chamber for a contact time of 24 h at 20 °C, the bacterial suspension was collected from the plates and transferred to 1 mL of saline with a mixture of LIVE/DEAD dyes (Invitrogen). The mixture contains two types of dyes (propidium iodide and Syto 9 Green (1:1)) which stain cells with an intact cell membrane (live) green and cells with cell membrane damage (dead) red-orange. After 20 min, samples were filtered through black polycarbonate membranes with a 0.22 μm pore size (EMD Millipore, Billerica, MA, USA), to separate the cells from the reaction mixture. Filters together with bacterial cells were then observed under a Nikon Eclipse E200 fluorescence microscope (Nikon Instruments, Tokyo, Japan). Then, color micrographs were taken using a Nikon Eclipse E200 with DS-Fi1 digital microscope camera and the NISElements F3.0 software package (Nikon Instruments, Tokyo, Japan). The number of cells on the micrographs was digitally counted using the image analyzer MultiScan Base v.14 (Computer Scanning Systems Ltd., Warsaw, Poland), and then expressed as a number of cells per milliliter.

## 3. Results and Discussion

### 3.1. Color and Transparency

**Figure 1:**
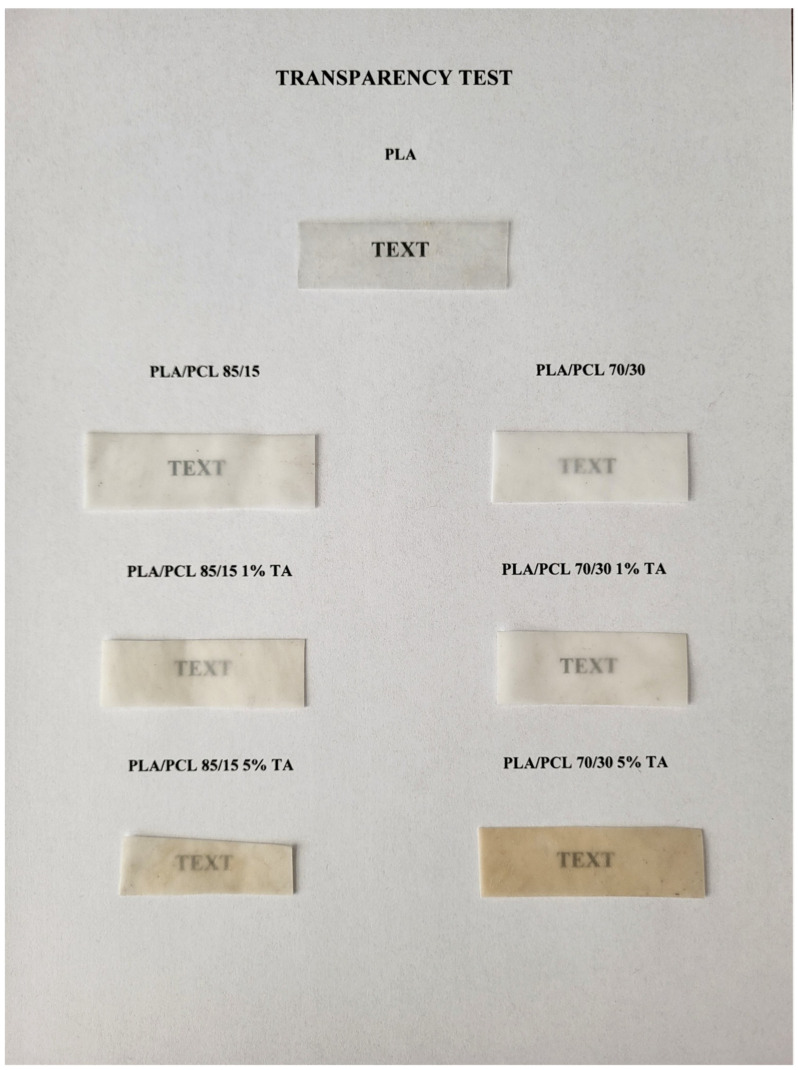
Color and transparency of the tested materials.

The color and transparency of polymeric materials intended for packaging are undoubtedly important quality parameters of these materials, which play a major role in the consumer’s choice. In connection with the above-mentioned analyses, a visual assessment of the color and transparency of the obtained polymeric materials was carried out (Figure 1), as well as an experimental determination of the color in the standardized color space of the CIELab system and transmittance at 550 nm wavelength, as well as changes of those parameters (Table 2).

Pure PLA is characterized by a lack of color and high transparency. Adding a second immiscible polymer—PCL—to PLA causes a change in color to white, and the resulting mixture loses its transparency. This effect is greater the higher the PCL content in the mixture. The observed change results from introducing a polymer with a higher degree of crystallinity to the mixture. The crystalline phase of PCL refracts light inside the material, giving a haze effect. Adding tannic acid to the polymer matrix causes a clear change in the color of the obtained materials. The change in the color of the tested materials towards yellow results from the color of tannic acid. A slight improvement in the transparency of the materials can be seen after adding tannic acid. This is especially visible in the case of samples containing a larger amount of the PCL phase. The text placed under the sample is more visible than in the case of PLA/PCL 70/30 1% TA and PLA/PCL 70/30 5% TA samples. This suggests that tannic acid dissolves/homogenizes better in the PCL phase (this was confirmed in other studies presented in the article).

The visual evaluation of the color and transmittance of the obtained materials and its changes are confirmed by the obtained numerical results.

The change in the color of the materials towards yellow, visible to the naked eye, was confirmed by the numerical values. With an increase in the tannic acid concentration, the b component increases, which reflects a yellow color in the CIELab space. The introduction of the second polymer phase in the form of PCL in the amount of 15 or 30 wt.% to the PLA matrix resulted in a ΔE value above 5. According to the principles of the CIELab color system, such a value means that the observer has the impression of two different colors [36]. ΔE values above 5 were also obtained for samples containing 5 wt.% tannic acid by mass. Therefore, also in the case of these materials, the observer sees two clearly different colors. Lower ΔE values were obtained for samples containing 1% tannic acid by mass. For PLA/PCL 85/15 1% TA and PLA/PCL 70/30 1% TA samples, the ΔE values were 4.28 and 3.77, respectively. However, according to CIELab rules, the observer looking at the two materials notices a clear difference in colors.

The visual changes in transparency of the tested materials were confirmed analytically. The wavelength of visible light to which the human eye is sensitive is between 380 and 780 nm. The light transmission of a good transparent material is expected to be very high in this range, and the maximum vision corresponds to a wavelength of 550 nm [32]. The assessment of the transparency of a given material can therefore be based on this value. Therefore, the analysis of the spectral results was based primarily on the transmittance value at a wavelength of 550 nm. However, the transmittance of the entire range of visible light is equally important, which is particularly important in terms of possible protection of stored food products against radiation

**Figure 2:**
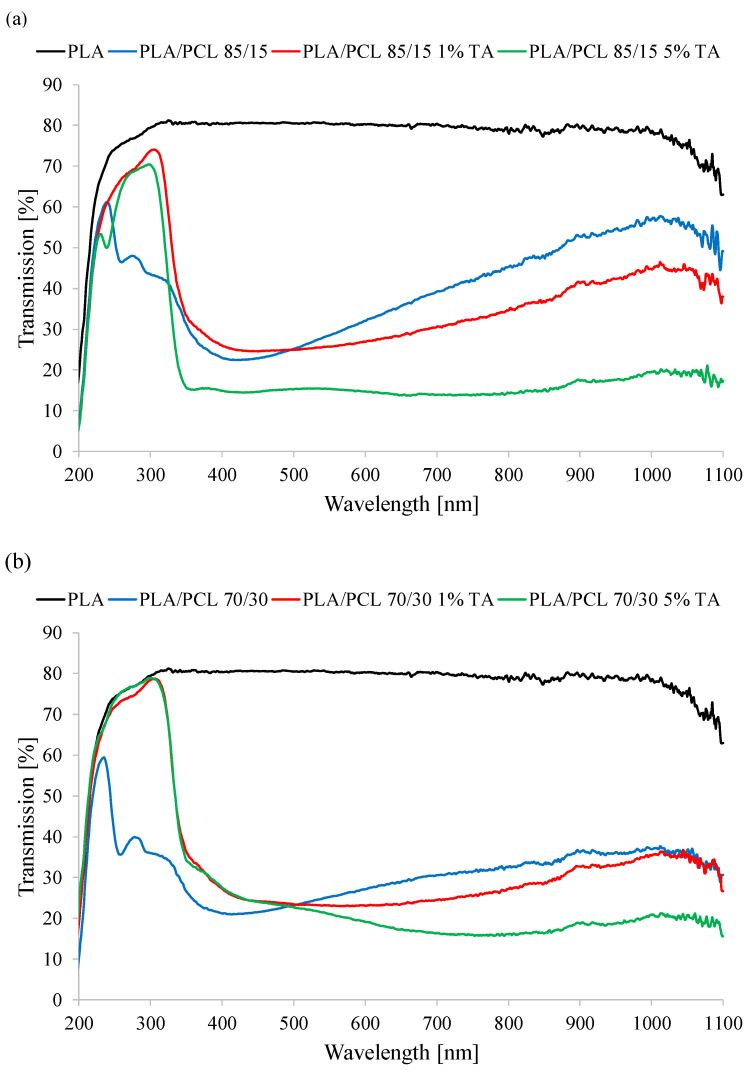
UV–Vis spectra of the tested materials.

The results of the UV–Vis transmittance analysis indicate that both the presence of the PCL phase and the addition of tannic acid significantly affect the visible light transmittance of the studied samples (Figure 2). The incorporation of PCL into the PLA matrix leads to a substantial decrease in transmittance at 550 nm wavelength, which may be attributed to differences in the refractive index and morphological changes within the polymer blend. These changes are due to the presence of the crystalline PCL phase in the mixture. Pure PLA exhibits high light transmittance at 80.3%, whereas the addition of 15% PCL results in a sharp decline to 28.4%. Increasing the PCL content to 30% leads to a further, albeit less pronounced, reduction in transmittance to 25.2%.

An additional factor affecting the optical properties of the materials is the presence of tannic acid. The introduction of this additive at a concentration of 1 wt.% further reduces visible light transmittance, which is particularly evident in PLA/PCL 85/15, where transmittance decreases to 25.7%, and in PLA/PCL 70/30, where it reaches 23.1%. At a higher tannic acid content (5 wt.%), this effect intensifies, leading to a further reduction in transmittance to 15.3% and 21.0%, respectively. These results suggest that tannic acid acts as an absorption-enhancing agent, likely due to its chromophoric properties and interactions with the polymer matrix, resulting in structural modifications of the material.

Although tannic acid typically absorbs ultraviolet (UV) radiation, primarily in the range of approximately 270–280 nm due to the presence of aromatic rings, under specific conditions, such as oxidative processes occurring during processing, it may give rise to colored products (e.g., quinones) that absorb within the visible spectrum, particularly around 550 nm, resulting in a decrease in transmittance in this region [37]. Additionally, tannic acid may exhibit limited miscibility with PLA/PCL, leading to the formation of micro- or submicron-sized aggregates within the polymer matrix. These aggregates increase optical turbulence, thereby reducing transparency. Furthermore, the presence of such aggregates may result in the formation of phase boundaries; differences in the refractive indices between phases can cause light scattering, which further diminishes transmittance.

Both the increased PCL content and the addition of tannic acid contribute to a decrease in all visible light spectrum transmittance (380–780 nm). This effect is particularly pronounced at higher tannic acid concentrations, indicating its strong light-absorbing properties. These findings are of particular relevance for applications requiring materials with reduced optical transparency, such as biodegradable protective barriers or packaging materials with controlled visible light permeability.

### 3.2. Microscopic Analysis

The SEM images of fractures obtained as a result of impact tests of the materials are shown in Figure 2.

**Figure 3:**
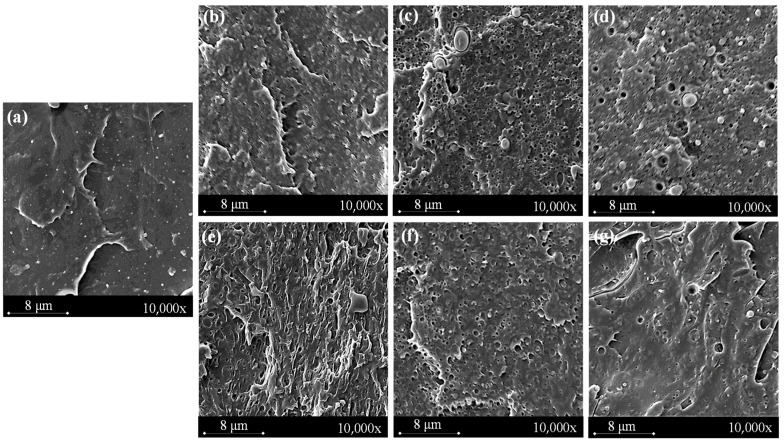
SEM images of sample fracture: (**a**) PLA, (**b**) PLA/PCL 85/15, (**c**) PLA/PCL 85/15 1% TA, (**d**) PLA/PCL 85/15 5% TA, (**e**) PLA/PCL 70/30, (**f**) PLA/PCL 70/30 1% TA, (**g**) PLA/PCL 70/30 5% TA.

Figure 3a shows a smooth microstructure with no irregularities, indicating the brittle nature of neat PLA. Detailed analysis of images of PLA/PCL (Figure 3b,e) mixtures indicates many dropout traces and visible boundaries between PLA and PCL in the impact fracture surface of the blends. The introduction of the PCL phase into the PLA matrix results in a change in the fracture behavior towards a more ductile character, which is particularly evident in the case of the PLA/PCL 70/30 sample (Figure 3e).

The presence of TA in the blend alters the nature of the observed fracture, particularly at a PCL phase content of 30 wt%. The fracture surface of the PLA/PCL 70/30 TA 5% sample more closely resembles that of pure PLA, indicating that TA influences the fracture behavior of the investigated materials. SEM images further confirm the differences in TA solubility depending on the PCL phase content in the blend. Image analysis clearly shows that in samples containing 15 wt.% PCL, the number of TA particles visible is significantly higher than in those with 30 wt.% PCL, which also helps to explain the observed change in the transparency of the studied materials.

### 3.3. Water Vapor Permeability

**Figure 4:**
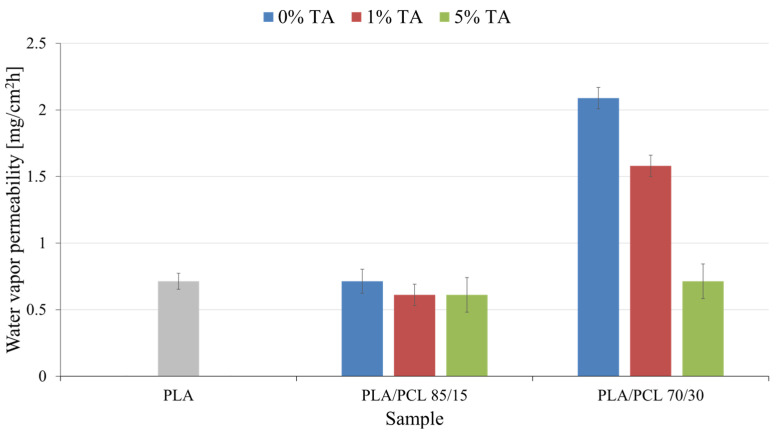
Water vapor permeability of the tested materials.

Water vapor permeability in polymeric materials used in the packaging industry is a key parameter because it affects the durability and safety of stored products. Permeability that is too high can lead to moisture absorption, which can cause content degradation, reduced quality or accelerated spoilage. On the other hand, permeability that is too low can impede gas exchange, which is particularly important for products that require specific storage conditions, such as food or medicines. Appropriate water vapor permeability therefore ensures optimal storage conditions and longer product durability. The results of water vapor permeability of the tested materials are shown in Figure 4.

The conducted studies revealed a varied water vapor permeability depending on the composition of the examined materials. Pure PLA exhibits a permeability of 0.71. The addition of 15% PCL in the PLA/PCL 85/15 blend does not cause a significant change in this value, which remains at 0.71. The introduction of 1 wt.% or 5 wt.% TA into the same blend results in a decrease in permeability to 0.61, suggesting that the presence of TA limits the diffusion of water molecules through the material.

Increasing the PCL content to 30% in the PLA/PCL 70/30 blend leads to a significant increase in water vapor permeability, reaching a value of 2.09. This phenomenon can be explained by the looser polymer structure and increased material flexibility, which facilitate the transport of water vapor. The addition of 1 wt.% TA to this blend results in a reduction in permeability to 1.58, while an increase in tannic acid content to 5 wt.% leads to a further decrease in permeability to 0.71.

The observed changes may result from several factors. PCL, being more flexible and hydrophobic than PLA, influences the polymer structure, and its higher content promotes increased water vapor permeability. TA, due to its ability to form hydrogen bonds, may seal the material structure, limiting the transport of water molecules. A possible cause of the reduction in water vapor permeability may also be the effect of water particle absorption by the tannic acid present in the material. It can also be assumed with great probability that the observed changes in permeability are caused by the simultaneous occurrence and overlap of the phenomena described above. A particularly significant effect is the synergistic interaction between PCL and TA in PLA/PCL 70/30 blends, where the addition of TA leads to a substantial reduction in permeability. It can be assumed that TA acts as a factor reducing the free volume of the polymer, thereby restricting water vapor diffusion.

### 3.4. Processing Properties

**Figure 5:**
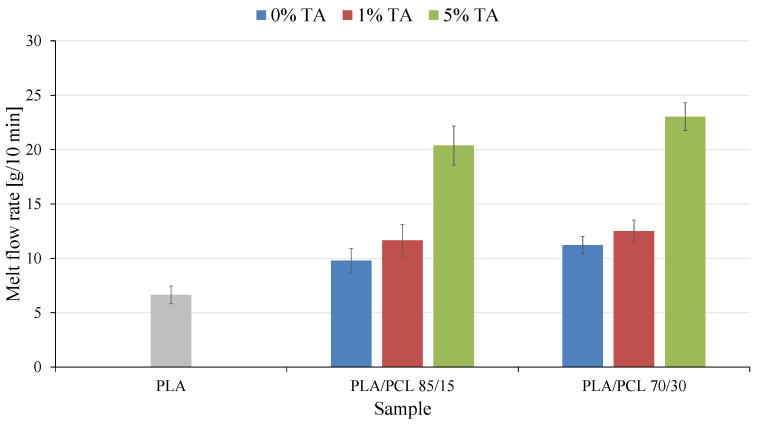
Mass flow rate of the tested materials.

Mass flow rate (MFR) is a parameter that allows for the assessment of the susceptibility of new polymer materials to a specific processing method. MFR tests were carried out, the results of which are presented in Figure 5.

First, the effect of PCL addition on the melt flow rate of the new polymer materials was assessed. The introduction of the second polymer phase PCL in the amount of 15 or 30 wt.% to PLA increased the MFR value by 3.2 and 4.9 g/10 min, respectively, which was an increase of 48 and 74% compared to the value of pure PLA. The increase in the MFR of the PLA/PCL mixture is mainly related to the significantly higher value of this parameter in the case of PCL. The MFR index can be defined as “point viscosity”. Therefore, the visible change in MFR is caused by the large difference in the viscosity of these two polymers. The differences in viscosity can be attributed to the large difference in the melting temperature between PLA (i.e., about 150 °C) and PCL (i.e., about 60 °C). This clear difference in viscosity between PLA and PCL significantly affects the viscosity of the blends and their processability [38].

Much more important from the point of view of the presented studies was the effect of tannic acid on the processing properties of the obtained polymer mixtures. Regardless of the PCL phase content in the tested polymer mixture, the addition of tannic acid caused an increase in the MFR of the obtained materials. This increase was higher when a higher amount of tannic acid was present in the material. The introduction of 1 wt.% of tannic acid caused an increase in the MFR of 20% for the PLA/PCL 85/15 1% TA sample and of 12% for the PLA/PCL 70/30 1% TA sample. In turn, as a result of the introduction of 5 wt.% of tannic acid, the MFR values of the obtained materials increased by 108% for the PLA/PCL 85/15 5% TA sample and by 105% for the PLA/PCL 70/30 5% TA sample.

According to the literature [39,40], polyphenols can weaken intermolecular interactions between polymer chains by moving them apart in space or by directly interacting with these chains as a result of reactions between tannic acid and the polymers contained in the mixture. This causes an increase in the mobility of polymer chains, which is reflected in an increase in the melt flow rate.

### 3.5. Mechanical Properties

The mechanical properties of polymer materials are of great importance in terms of their potential practical applications. The conducted research determined selected mechanical parameters that are important in the packaging industry, i.e., tensile strength, elongation at break and impact strength.

**Figure 6:**
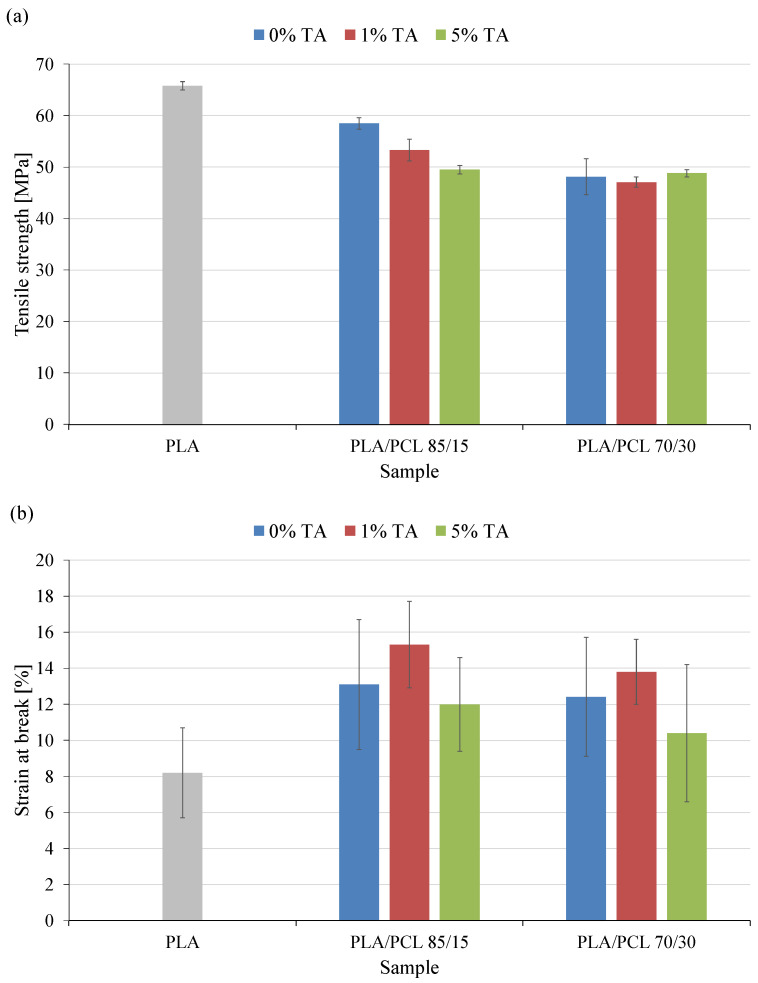
Results of static tensile strength tests: (**a**) tensile strength, (**b**) strain at break.

The PLA/PCL blend is characterized by a lower tensile strength (σ_M_) than pure PLA (Figure 6a). The introduction of 15 wt.% of PCL into the PLA matrix reduced the σ_M_ value of the PLA/PCL 85/15 sample by 7.3 MPa. Increasing the PCL content in the blend caused an even greater decrease in σ_M_, i.e., by 17.7 MPa. The reduction in the tensile strength of PLA/PCL blends in relation to PLA is a consequence of the significantly lower mechanical strength of PCL [41].

As for the effect of tannic acid in the tested materials, the conducted studies have shown that it is significant only in the case of samples containing 15 wt.% of PCL. In the case of these materials, an increase in the content of tannic acid caused a decrease in tensile strength. 1 wt.% of tannic acid caused a decrease in the σ_M_ value of the PLA/PCL 85/15 1% TA sample by 5 MPa (10%) compared to the PLA/PCL 85/15 sample. A decrease in the σ_M_ value by 9 MPa (15%) was observed for the PLA/PCL 85/15 5% TA sample. The effect of tannic acid on tensile strength was not observed with a higher content of PCL. The PLA/PCL 70/30 samples had small values similar to each other regardless of the tannic acid content.

The observed decrease in tensile strength after the introduction of tannic acid is related to the plasticizing properties of this compound. It has previously been shown that the plasticizing effect of additives introduced to the polymer affects the mechanical properties [42]. Since the low-molecular plasticizer behaves like a solvent when mixed with the polymer, this leads to a decrease in cohesion between macromolecular chains. The previously mentioned decrease in the interaction between polymer chains, improving the mobility of polymer chains, leads to a significant decrease in tensile strength, which is observed in the obtained test results.

The decrease in tensile strength is accompanied by an increase in elongation at break (ε_B_). The introduction of the more flexible PCL to PLA causes an increase in this parameter. Regardless of the amount of PCL in the mixture, the increase in ε_B_ after adding this polymer was about 60%. Analyzing the average values of ε_B_, it can be seen that tannic acid also caused some changes in the elongation at break of the tested materials. A concentration of 1 wt.% tannic acid caused an increase in ε_B_ of about 2% for both mixtures compared to the mixtures without this compound. Increasing the tannic acid concentration to 5 wt.% caused a decrease in ε_B_ of the mixtures by about 2%. It can therefore be seen that at a lower concentration of tannic acid, the plasticizing properties of this compound become visible. At a higher concentration, however, phenomena related to disruptions in the structure of the cross-section of materials caused by plasticizer particles in the polymer matrix probably begin to dominate. This causes the formation of local stresses, which contribute to faster cracking of the materials and a reduction in the final deformations. Although a certain common trend of changes is visible in the graph (Figure 6b), the obtained high values of the standard deviation (SD) allow us to conclude that the observed changes are not significant.

PLA/PCL blends with tannic acid offer a compromise between the strength of PLA and the flexibility of PCL. Compared to other polymers, the obtained blends occupy an intermediate position between rigid materials (PET, PVC) and flexible ones (PE, PP). In comparison with other polymers [43], polyethylene (PE) has a tensile strength of 10–30 MPa and a high elongation at break (100–1000%), polypropylene (PP) exhibits a tensile strength of 25–40 MPa and elongation of 100–600%, poly(ethylene terephthalate) (PET) has a tensile strength of 50–80 MPa and elongation of 50–150%, and rigid poly(vinyl chloride) (PVC) demonstrates a tensile strength of 40–80 MPa and elongation of 20–40%. These properties of the obtained blends may be advantageous for packaging applications, where moderate mechanical resistance and deformation capability are required.

**Figure 7:**
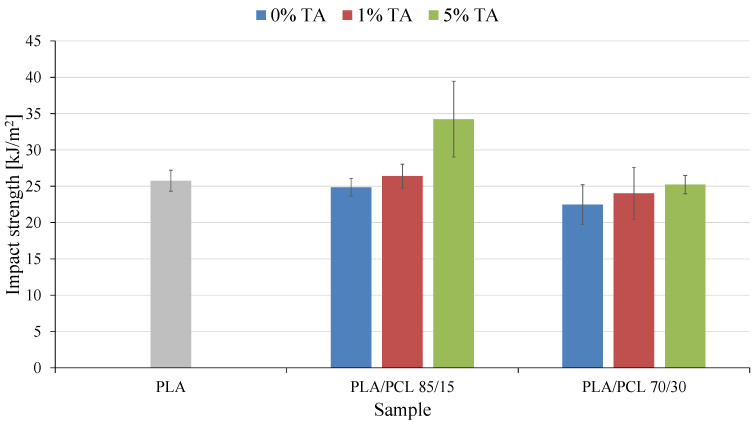
Impact strength of the tested materials.

The last mechanical parameter determined was impact strength (Figure 7). In many cases, impact properties are the most important mechanical data in practical applications, because they determine the ability to deform at high strain rates. Indeed, materials showing a large strain at break during the rather slow tensile test procedure do not necessarily show the same ability to deform at the high strain rates used in the impact test [44].

The introduction of PCL to PLA affected the impact strength (u_a_) of the obtained mixture only at 30 wt.% of this polymer. The observed decrease in u_a_ of the PLA/PCL 70/30 sample was 3.3 kJ/m^2^ (13%). The observed effect is not a typical result of introducing PCL to PLA. Usually, an increase in impact strength is observed as a result of introducing a more flexible PCL phase to the mixture.

A decrease in impact strength following the incorporation of 30% polycaprolactone (PCL) into a polylactide (PLA) matrix can be attributed to several interrelated factors. Although PCL is a more ductile and flexible polymer compared to PLA, and its addition is generally expected to enhance the toughness of polymer blends, the observed reduction in impact resistance may result from limited compatibility between the two components. PLA and PCL are only partially miscible, and without the use of an appropriate compatibilizer, the blend may exhibit poor interfacial adhesion. This can lead to the formation of phase-separated structures with weak interphase bonding, which in turn promotes crack initiation and propagation under dynamic loading conditions, thereby reducing the material’s overall impact strength. Higher PCL content in the mixture may exacerbate this phenomenon.

Furthermore, the morphology of the blend plays a critical role in determining mechanical performance. At a PCL content of 30%, the system is likely to form dispersed PCL domains within the PLA matrix. If these domains are not finely and uniformly distributed, or if their size exceeds a critical threshold, they may act as stress concentrators rather than energy-dissipating regions. This unfavorable morphology can significantly impair the material’s ability to withstand sudden impact forces. Additionally, the inherent crystallization behavior of PCL may contribute to the problem. Due to its higher crystallization rate compared to PLA, PCL can form semi-crystalline domains upon cooling, which may act as crack initiation sites under mechanical stress.

In [45] it was shown that in many cases the addition of PCL had a negative effect on the impact strength of the obtained mixture, which is related to, among others, the degree of crystallinity of the PLA phase and the grain size of the PCL phase. Explaining the influence of the PCL phase on the impact strength of the tested materials was not the aim of this study.

The main objective of the presented research was to determine the effect of tannic acid on the impact strength of the obtained materials. During the research, a positive effect of tannic acid on the impact strength of the tested materials was observed. With the increase of this compound in the mixtures, the u_a_ value increased. In the case of most materials the changes were not large and did not exceed 3.0 kJ/m^2^. The exception was the PLA/PCL 85/15 5% TA sample, where the obtained increase in u_a_ was 9.3 kJ/m^2^.

The plasticizing properties of tannic acid are thus once again revealed. Materials become more elastic. The increased mobility of macromolecules means that they can absorb greater amounts of energy, which increases the energy needed to break them.

### 3.6. Thermomechanical Properties

One of the characteristic features of polymeric materials is that their mechanical properties strongly depend on temperature. With changes in this parameter, the mechanical properties of polymeric materials can change drastically. The change in mechanical properties as a function of temperature is therefore important in terms of the possible use of new materials in the packaging industry. Very often, the conditions of use and storage of packaging are extreme and occur in a wide temperature range.

**Figure 8:**
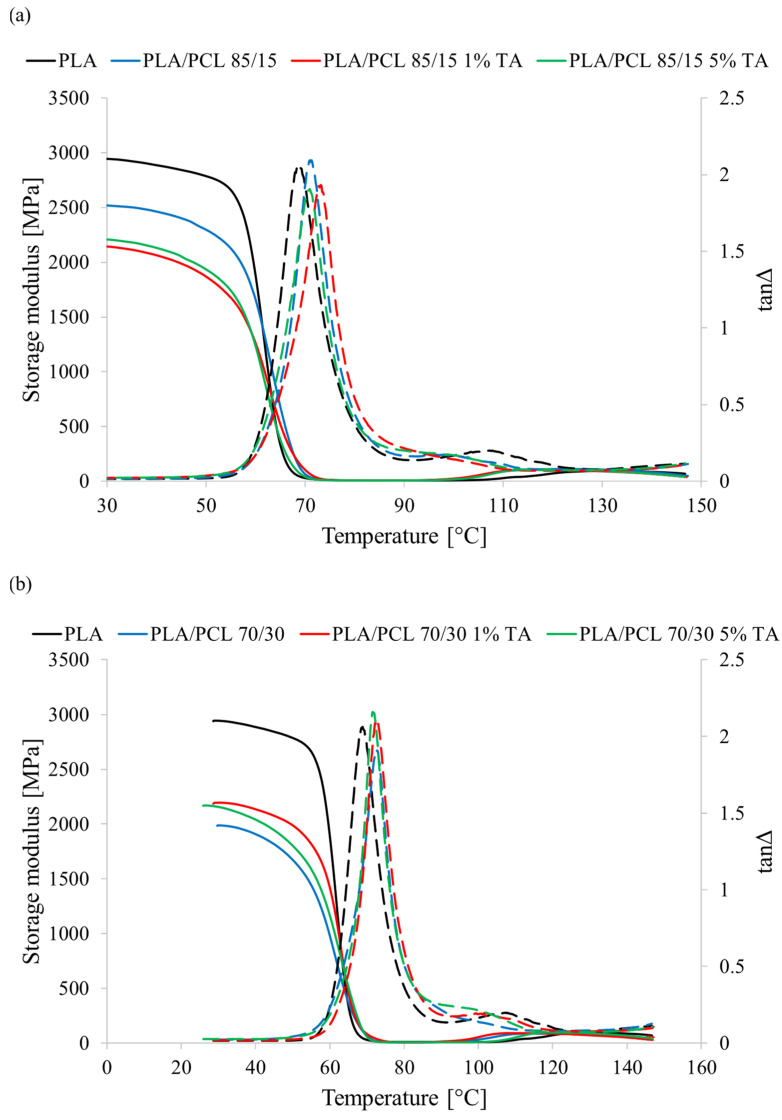
Thermomechanical curves of (**a**) PLA/PCL 85/15 samples, (**b**) PLA/PCL 70/30 samples; solid line—storage modulus, dashed line—tanΔ.

Table 3 presents the results of measurements of thermomechanical properties (storage modulus) of the tested materials. Figure 8 presents the thermomechanical curves of the tested materials showing changes in storage modulus (E’) as a function of temperature.

Pure PLA is characterized by quite high stiffness and its storage modulus at 30 °C (E’30) is 2944 MPa. The recorded course of the thermomechanical curve as a function of temperature of the PLA sample is typical for this polymer. One can observe a sharp decrease in E’ in the range of 60 to 80 °C related to the glass transition of PLA, the value of which determined by the DMA method is 68.8 °C. Then, a slight increase in E’ is also visible due to the cold crystallization process of this polymer. This phenomenon occurs in the range of 110 to 150 °C.

The introduction of PCL does not affect the thermomechanical curves in the entire range of applied temperatures. We still observe changes similar to those in the case of pure PLA (glass transition, cold crystallization). However, the addition of a second polymer (PCL) causes a decrease in the E’ value in the obtained mixtures. The decrease in stiffness is greater the higher the content of the PCL phase. After adding 15 wt.% PCL the E’30 value decreased by 423 MPa compared to the value obtained for pure PLA. The addition of 30 wt.% PCL decreased the E’30 value by 959 MPa. The observed decrease is a typical phenomenon after introducing a low-modulus material such as PCL into the mixture [46]. This is normal in the case of immiscible and incompatible polymer blends such as PLA/PCL, where certain properties of the blends are averaged out when compared to the properties of the individual polymers comprising the blend.

The introduction of PCL also influenced the glass transition temperature of the PLA phase determined by the DMA method. The T_g_ value increased by 2.5 °C for the PLA/PCL 85/15 sample and 3.6 °C for the PLA/PCL 70/30 sample. The increase in the glass transition temperature was the effect of the penetration of PCL macromolecules between PLA macromolecules, which slightly hindered their mobility. 

The effect of tannic acid on the storage modulus of the tested materials depended on the PCL phase content in the mixture. At 15 wt.% PCL phase, the addition of tannic acid caused a decrease in the E’30 value compared to the PLA/PCL 85/15 sample. A concentration of 1 wt.% of tannic acid caused a decrease in E’30 by 377 MPa. Increasing the tannic acid concentration to 5 wt.% slightly limited this decrease to 312 MPa. On the other hand, at 30 wt.% PCL phase, the addition of tannic acid caused an increase in the E’30 value compared to the PLA/PCL 70/30 sample. A concentration of 1 wt.% of tannic acid caused an increase in E’30 by 209 MPa. Increasing the tannic acid concentration to 5 wt.% slightly limited this increase to 168 MPa. Interestingly, however, regardless of the amount of the PCL phase in the mixture, the obtained E’30 values were very similar for materials containing tannic acid. This means that the stiffness of these materials at room temperature is very similar.

Moreover, in both cases of PCL phase content, the addition of tannic acid, regardless of its concentration, did not cause significant changes in the glass transition temperature determined by the DMA method.

### 3.7. Thermal Properties

Thermal properties, next to mechanical properties, are the second most important group of parameters in the case of processing and use of polymeric materials. As part of the study, the thermal parameters of the obtained materials were determined using thermogravimetry and differential scanning calorimetry techniques. The thermal parameters of the tested materials are listed in Table 4. Prior to the fabrication of the studied materials, the thermal stability of tannic acid was analyzed to verify its processability at the processing temperatures of the applied polymers. The recorded temperature corresponding to a 5% mass loss of tannic acid was 196.8 °C. However, the initial mass loss was not associated with the degradation of tannic acid but rather with the evaporation of moisture [47]. The main degradation stage of tannic acid begins at approximately 200–210 °C. This indicates that the processing temperatures used allow for the safe processing of tannic acid.

**Figure 9:**
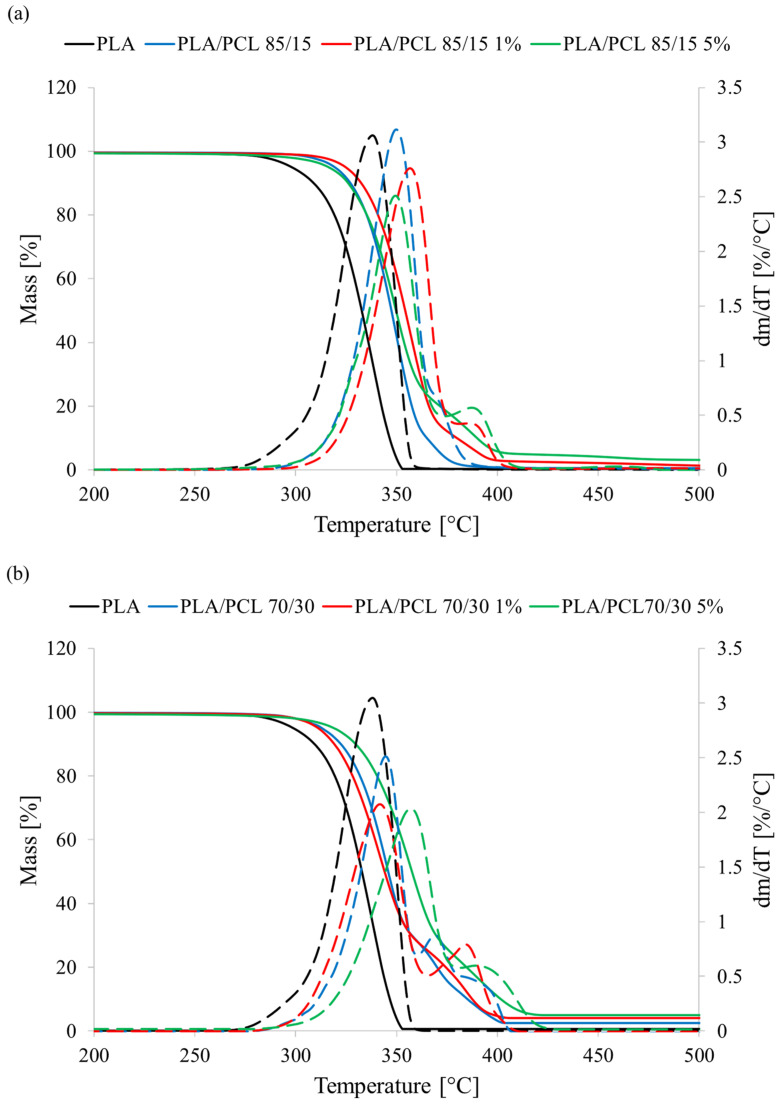
Thermogravimetric curves of samples (**a**) PLA/PCL 85/15, (**b**) PLA/PCL 70/30; continuous line—mass, dashed line—dm/dT.

The first thermal parameter of the tested materials was thermal resistance (T_d_) determined by the thermogravimetric method. The PLA sample had a T_d_ of 297.2 °C. PCL is characterized by a higher thermal resistance than PLA (360–370 °C vs. 290–320 °C) [48,49], hence the T_d_ values of materials containing this polymer were higher than those of pure PLA (Figure 9). The presence of 15 wt.% PCL in the mixture increased the thermal resistance of the PLA/PCL 85/15 sample by 21.7 °C (8%). In the case of the material containing 30 wt.% PCL, the increase was slightly lower, as the T_d_ of the PLA/PCL 70/30 sample increased only by 14.5 °C (5%). Tannic acid did not negatively affect the thermal resistance of the produced polymer blends. However, no clear trend was found that could determine the effect of this compound on the T_d_ of PLA/PCL blends. In the PLA/PCL 85/15 samples, the highest T_d_ value was characteristic for the material containing 1% tannic acid. In the PLA/PCL 70/30 samples, the material containing 5% tannic acid demonstrated the highest thermal resistance. The most important result of these studies is, however, that the introduction of tannic acid does not cause significant changes in thermal resistance, at least up to temperatures that would prevent the processing of the tested materials. The obtained T_d_ values significantly exceed the typical extrusion and injection temperatures of the polymers used. The observed changes (one way or the other) are therefore of no great significance.

**Figure 10:**
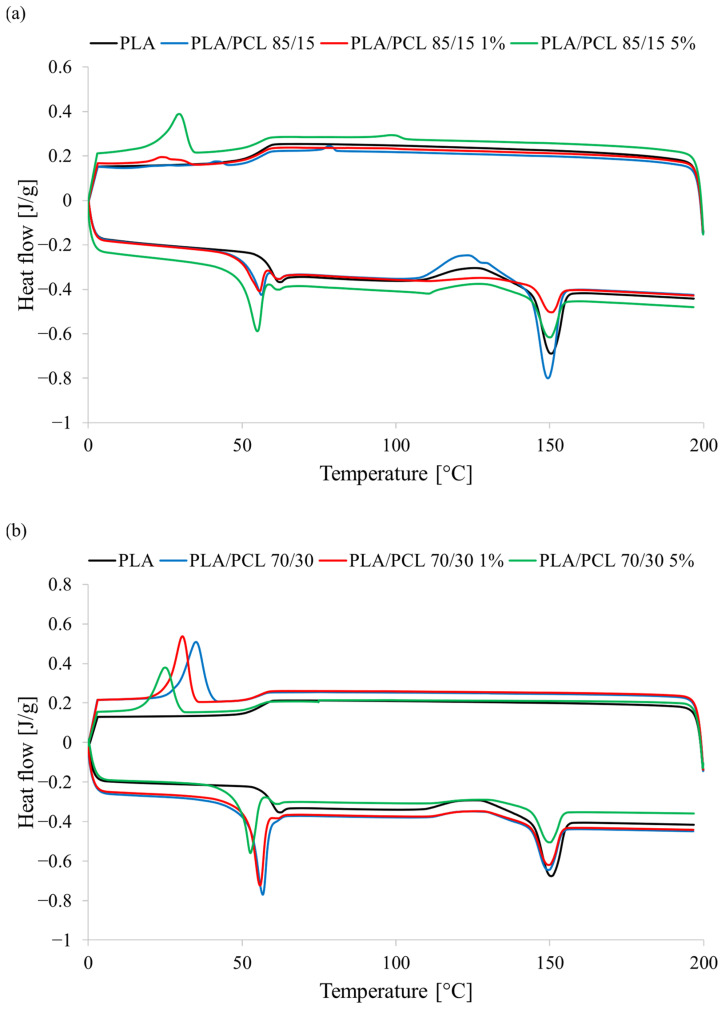
Thermal curves of samples (**a**) PLA/PCL 85/15, (**b**) PLA/PCL 70/30; exo up.

In the processing of polymeric materials, it is important to know the occurrence and kinetics of phase transitions of the processed materials. For these reasons, phase transitions occurring in the tested materials during the cooling and heating processes were determined and analyzed (Figure 10).

Due to the overlap of the PLA glass transition temperature (~55 °C) with the PCL melting temperature (55–60 °C), the value of the former was determined from the cooling curve. The occurrence of the supercooling phenomenon in the case of PCL lowered the temperature of the crystallization process, due to which the phase transitions of these polymers were separated. The T_g_ of PLA was 56.4 °C. Adding PCL to the PLA matrix did not cause significant changes in this parameter regardless of the amount of this polymer introduced. This is a different situation than in the case of the glass transition temperature determined by the DMA method. However, this results from the different characteristics of determining this parameter using these research methods. In the case of DSC, the glass transition temperature is measured based on thermal effects. In DMA, the glass transition temperature is determined based on changes in mechanical parameters.

The discrepancy in the glass transition temperature determined by DSC and DMA arises from the fundamentally different principles underlying these techniques and their varying sensitivities to the physical changes occurring during the glass transition [50]. DSC measures the heat flow associated with temperature changes and detects the glass transition as a subtle shift in the material’s heat capacity. In contrast, DMA assesses the mechanical properties of the sample—storage modulus, loss modulus, and mechanical damping (tan δ)—in response to oscillatory deformation. Since the glass transition involves changes in the mobility of polymer chain segments, and DMA is particularly sensitive to such molecular motions, it often captures the transition more distinctly and at a higher temperature than DSC.

Moreover, the observed difference is influenced by the different time and frequency scales inherent to each method. DSC measurements are typically conducted under steady heating without applying mechanical stress, whereas DMA involves subjecting the material to dynamic mechanical loading at specific frequencies. At higher frequencies, the material’s molecular segments require more thermal energy (i.e., higher temperature) to respond, leading to an elevated apparent T_g_ in DMA. In most cases, lower values of the glass transition temperature are obtained in the DSC method, which was also observed in the presented studies.

The introduction of tannic acid did not cause any significant changes in the glass transition temperature of the PLA phase. Although changes in T_g_ towards higher or lower temperatures were observed, they were not greater than 1.5 °C, so they cannot be considered significant. Tannic acid did not significantly affect other PLA phase transitions. Only a slight increase in the cold crystallization temperature (T_cc_) was observed, with a simultaneous decrease in the intensity of this process (ΔH_cc_) with increasing tannic acid concentration, regardless of the PCL phase content in the blend. This confirms the previously indicated limitation of macromolecular mobility by tannic acid, which results in a limited possibility of PLA chain ordering in the cold crystallization process. The effect of reducing the intensity of the cold crystallization process was a reduction in the intensity of the melting process of the formed PLA crystalline phase. At the same time, very similar enthalpy values of ΔH_ccPLA_ and ΔH_mPLA_ indicate that the input degree of crystallinity of the PLA phase in both mixed systems was close to zero and independent of the tannic acid content. The obtained results also allow us to state that the PLA phase occurs in the mixture in an amorphous form.

The influence of tannic acid was, however, greater in the PCL phase of the tested mixtures. The first visible effect of the influence of tannic acid on the phase transitions of PCL was a significant decrease in the crystallization temperature of T_cPCL_ of this polymer during cooling. The drop in crystallization temperature was greater the higher the content of this compound in the mixture. A concentration of 1 wt.% tannic acid decreased the T_cPCL_ of the PLA/PCL 85/15 1% TA sample by 12.0 °C compared to the PLA/PCL 85/15 sample. Increasing the tannic acid concentration to 5 wt.% further reduced the crystallization temperature of the PCL phase, as the T_cPCL_ of the PLA/PCL 85/15 5% TA sample was lower by 17.6 °C. A similar decreasing trend was observed for materials containing 30 wt.% PCL. A concentration of 1% tannic acid reduced the T_cPCL_ of the PLA/PCL 70/30 1% TA sample by 4.5 °C compared to the PLA/PCL 70/30 sample, and 5% tannic acid by 10.1 °C.

The changes in the crystallization process resulted in changes in the melting process of the PCL phase. First, the introduction of tannic acid to the PLA/PCL blend causes changes in T_mPCL_. At 15 wt.% of PCL, the decrease in this parameter was small—only 1.2 °C—even at the highest content of tannic acid. A much greater decrease in T_mPCL_ was observed at 30 wt.% of PCL. The T_mPCL_ value of the PLA/PCL 70/30 5% TA sample was lower by 4.1 °C compared to the PLA/PCL 70/30 sample. However, these were not as large changes as in the case of T_cPCL_.

Tannic acid also affected the degree of crystallinity of the PCL phase. At 15 wt.% PCL content in the mixture, the degree of crystallinity of the material containing 5 wt.% tannic acid (PLA/PCL 85/15 5% TA) calculated on the basis of ΔH_mPCL_ increased almost twofold compared to the PLA/PCL 85/15 sample. After increasing the PCL phase content to 30 wt.% in the tested polymer mixture, the effect of tannic acid is reversed. The introduction of this compound to the mixture causes a decrease in the degree of crystallinity of the PCL phase by a maximum of 1/3 of the value of the PLA/PCL 70/30 sample.

Based on the DSC studies, two main conclusions can be drawn. First, the greater changes in the PCL phase of the studied mixture may suggest that tannic acid accumulates more in the PCL phase of the obtained polymer mixtures. Second, it seems that tannic acid affects the kinetics of the PCL crystallization process more than the structure of the resulting crystalline phase. Tannic acid hinders the ordering of the PCL phase chains, which translates into lower crystallization temperatures. However, the structure of the resulting crystalline phase is very similar, as evidenced by the similar melting temperature values of the resulting PCL crystalline phase.

### 3.8. Antibacterial Properties

After conducting tests on processing, mechanical and thermal properties, which confirmed that the proposed materials have properties suitable for applications related to the packaging industry, the main assumption made for the new material was checked, i.e., biocidal properties in relation to microorganisms. Biocidal tests were performed in accordance with the research procedure presented in the paragraph “materials and methodology”. The reductions (R) of the bacterial strains of *Escherichia coli* and *Staphylococcus aureus* for the tested samples are listed in Table 5 and Table 6. According to a relevant standard [35], the reduction degree (R) relating to bacteria must be at least 2, if a biocidal agent is to be considered as effective.

**Figure 11:**
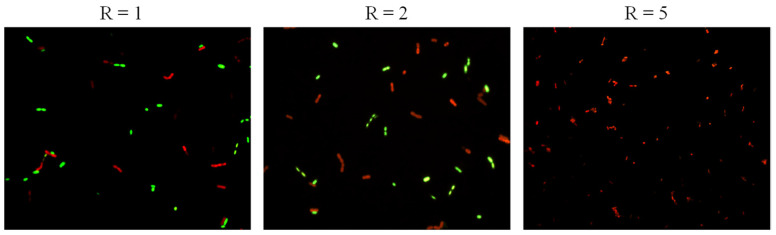
Epifluorescence microscopy images of samples with different reduction factor (R) for the example of PLA/PCL 85/15 1% (R = 1), PLA/PC 85/15 5% (R = 2), PLA/PCL 70/30 5% (R = 5) and *Escherichia coli* strain (ATCC8739); green—live cells; red—dead cells.

Based on the obtained results, only PLA/PC 85/15 5% TA (R = 2), PLA/PCL70/30 1% TA (R = 3) and PLA/PCL 70/30 5% TA (R = 5) have confirmed biocidal effects. It can therefore be seen that the most beneficial in terms of antibacterial effect is the use of a higher amount of tannic acid (Figure 11). Differences in the biocidal activity of the tested materials result from differences in the structure of the cells of the test strains. *S. aureus* are Gram-positive bacteria whose cell structures consist of a cell membrane and a thick cell wall composed of peptidoglycan. These bacteria do not produce any additional shields. *E. coli*, on the other hand, are Gram-negative bacteria whose cell wall is relatively thin, but is additionally surrounded by an outer membrane that makes it difficult for many substances to penetrate into the immediate vicinity of the wall and cell membrane. For this reason, many antibacterial or bactericidal agents have a weaker effect on Gram-negative bacteria.

The results of the biocidal tests indicate that the PLA/PCL 70/30 5% TA material has the most beneficial antibacterial properties. This material has shown high antibacterial effectiveness against both strains of bacteria used, and it is this material that has the greatest potential for use in the production of active food packaging.

The obtained results were compared with those reported in studies [12,13,14,15,16,17,18,19,20,21,22,23,24,25,26] concerning packaging materials with antibacterial properties, incorporating both inorganic and organic active substances. These studies describe the efficacy of metal and metal oxide nanoparticles, such as silver (Ag), zinc oxide (ZnO), copper oxide (CuO), and titanium dioxide (TiO_2_), which exhibit potent antimicrobial activity, often at lower concentrations than the TA used in the present work. For instance, silver nanoparticles embedded in a polymer matrix were able to reduce the *E. coli* population by over 5 log units within 24 h at a concentration of 0.5 wt% Ag. Similarly, zinc oxide nanoparticles achieved a 99.9% reduction of *S. aureus* at just 1% concentration, corresponding to an R value of approximately 3. In other studies, active packaging containing copper oxide nanoparticles led to a reduction of *E. coli* by around 4 log units after 6 h of contact at a 2 wt.% CuO content.

In contrast, nanostructures based on layered clays such as bentonite or montmorillonite typically require additional active substances to achieve significant antimicrobial effects. Nanocomposites comprising nanoclay and essential oils can achieve bacterial reductions in the range of 2–4 log units, depending on the type of oil and component concentrations. Their efficacy against Gram-negative bacteria such as *E. coli* is frequently lower compared to Gram-positive strains, which aligns with the results observed for PLA/PCL blends containing TA. For example, a system based on montmorillonite and thyme essential oil reduced *S. aureus* counts by 99.5% (R ≈ 2.3), whereas the reduction of *E. coli* was only 80% (R ≈ 0.7).

These studies also highlight the use of naturally derived substances, such as essential oils, which demonstrate high antimicrobial efficacy. The incorporation of 1.5 wt.% eucalyptus oil into a biopolymer matrix resulted in a 4.2 log unit reduction of *E. coli* within 24 h. Other oils, such as thyme or oregano, exhibit even stronger effects than TA at lower concentrations (0.5–2 wt.%). However, these oils are often limited by their intense aroma and low thermal stability, which can restrict their use in industrial processing. These limitations may adversely affect the sensory properties of the packaging and the food stored within, reducing their overall applicability.

Chitosan and its composites are also extensively studied as alternatives to conventional antimicrobial agents. Chitosan films containing 2 wt.% green tea extract reduced *S. aureus* by 99.8% (R ≈ 2.7) and *E. coli* by 90% (R ≈ 1). These systems demonstrate high efficacy against both bacterial types; however, their activity is pH-dependent, which may restrict their practical utility. In recent years, increasing attention has also been given to enzymes such as lactase and targeted enzymes that can selectively degrade microbial cell walls. The application of lactase in the active layer of packaging materials may result in a reduction of Gram-positive bacteria by up to 5 log units under laboratory conditions.

Against the backdrop of the aforementioned solutions, tannic acid—as a natural phenolic antibacterial compound—demonstrates moderate yet distinct antimicrobial activity in PLA/PCL composites, particularly at higher concentrations. Although a 5 wt.% addition of TA was required in the tested materials to achieve high efficacy against both bacterial types, its advantages include low toxicity, biodegradability, and good compatibility with the polymer matrix. In light of the above data, the PLA/PCL 70/30 blend with 5 wt.% TA can be considered a material with significant application potential for the development of active food packaging, offering a favorable balance between microbiological performance, user safety, and environmental sustainability.

## 4. Conclusions

The paper presents the test results of selected processing, mechanical and thermal properties of a polylactide (PLA) and polycaprolactone (PCL) mixture containing tannic acid. The purpose of introducing tannic acid into the polymer matrix was to improve the miscibility of these polymers and to give the obtained material biocidal properties. Based on the conducted tests, it was found that adding tannic acid to the PLA/PCL mixture causes a clear change in the color of the obtained materials towards yellow, which results from the color of tannic acid. The color change is accompanied by a slight improvement in the transparency of the materials, especially those containing a higher content of the PCL phase. This suggests that tannic acid dissolves/homogenizes better in the PCL phase.

The study demonstrates that water vapor permeability of PLA-based materials varies depending on composition. While the addition of 15% PCL does not significantly affect permeability, increasing PCL content to 30% leads to a substantial rise due to structural loosening. However, incorporating tannic acid (TA) reduces permeability, likely due to hydrogen bonding and free volume reduction. The most pronounced effect is observed in PLA/PCL 70/30 blends, where TA significantly limits water vapor diffusion. These findings highlight the interplay between polymer flexibility and TA-induced structural modifications in controlling material permeability.

The obtained results did not confirm the compatibilizing effect of tannic acid, because the obtained mechanical properties were slightly worse than the materials without the addition of this compound. Tannic acid acted more like a plasticizer, because its addition caused an increase in the melt flow rate of the obtained materials. This increase was higher in the case of a higher content of tannic acid in the material. The maximum increase in the melt flow rate exceeded 100% in the case of some materials. The plasticizing effect of tannic acid was also observed in the mechanical properties of the obtained composites. The presence of tannic acid caused a decrease in tensile strength (max. 15%), an increase in elongation at break (max. 90%) and an increase in impact strength (max. 36%).

Tannic acid did not negatively affect the thermal resistance of the produced polymer blends, the glass transition temperature of the polylactide phase, or the other phase transformation temperatures of polylactide. Only a slight increase in the cold crystallization temperature of this polymer was observed, with a simultaneous decrease in the intensity of this process with the increase in the content of tannic acid in the blend. The effect of tannic acid was, however, greater in the case of the polycaprolactone phase of the studied blends. The first visible effect of the influence of tannic acid on the phase transitions of polylactide was a significant decrease in the crystallization temperature of this polymer during cooling. The drop in crystallization temperature was higher the higher the concentration of tannic acid in the blend. Tannic acid also affected the degree of crystallinity of the polycaprolactone phase.

The obtained mixture was characterized as having biocidal properties against two strains of bacteria *Escherichia coli* (ATCC 8739) and *Staphylococcus aureus* (ATCC 6538P). Antibacterial properties together with acceptable processing, mechanical and thermal properties indicate that the presented polymer material may be a potential material for the production of active food packaging. In comparison to the other approaches found in the literature, tannic acid exhibits moderate yet clearly discernible antimicrobial activity in PLA/PCL composites, particularly at elevated concentrations. While a 5 wt.% loading of tannic acid was necessary to achieve substantial efficacy against both Gram-positive and Gram-negative bacteria, its favorable attributes—low toxicity, biodegradability, and good compatibility with the polymer matrix—enhance its applicability. Based on the presented findings, the PLA/PCL 70/30 blend incorporating 5 wt.% tannic acid may be regarded as a promising candidate for the development of active food packaging materials, offering a well-balanced combination of antimicrobial effectiveness, user safety, and environmental sustainability.

## Figures and Tables

**Table 1:** The designation and composition of samples.

Sample	PLA [%]	PCL [%]	TA [%]
PLA	100	-	-
PLA/PCL 85/15	85	15	0
PLA/PCL 85/15 1% TA	85	15	1 *
PLA/PCL 85/15 5% TA	85	15	5 *
PLA/PCL 70/30	70	30	0
PLA/PCL 70/30 1% TA	70	30	1 *
PLA/PCL 70/30 5% TA	70	30	5 *

* The concentration of tannic acid was related to the mass of the PLA/PCL mixture.

**Table 2:** Color measurement in CIELab space and transmittance at 550 nm wavelength determined by UV–Vis spectroscopy.

Sample	CIELab Values				Uv–Vis
	L	a	b	ΔE	T_550nm_ [%]
PLA	74.03	−0.11	6.22	-	80.3
PLA/PCL 85/15	73.85	−2.50	1.24	5.52	28.4
PLA/PCL 85/15 1% TA	73.14	−0.41	4.92	4.28	25.7
PLA/PCL 85/15 5% TA	73.87	1.88	6.76	7.05	15.3
PLA/PCL 70/30	76.98	−1.77	1.06	6.18	25.2
PLA/PCL 70/30 1% TA	75.99	−0.20	4.34	3.77	23.1
PLA/PCL 70/30 5% TA	74.84	1.40	6.83	6.92	21.0

**Table 3:** Storage modulus values of the tested materials at different temperatures along with the glass transition value determined by the DMA method.

Sample	E’_30_ [MPa]	E’_50_ [MPa]	E’_70_ [MPa]	E’_100_ [MPa]	E’_130_ [MPa]	T_gDMA_ [°C]
PLA	2944.0	2787.0	29.9	7.0	95.3	68.8
PLA/PCL 85/15	2521.0	2299.0	64.6	21.1	107.3	71.3
PLA/PCL 85/15 1% TA	2144.0	1871.0	105.0	23.1	88.0	72.2
PLA/PCL 85/15 5% TA	2209.0	1936.0	40.3	16.6	93.0	71.0
PLA/PCL 70/30	1985.0	1670.0	63.3	32.4	71.7	72.4
PLA/PCL 70/30 1% TA	2194.0	1988.0	83.4	54.9	68.9	71.2
PLA/PCL 70/30 5% TA	2153.0	1798.0	52.3	9.3	87.0	71.4

**Table 4:** Thermal parameters of tested materials.

Sample	T_d_ [°C]	DSC—Cooling			DSC—Heating					
		T_gPLA_ [°C]	T_cPCL_ [°C]	ΔH_cPCL_ [J/g]	T_mPCL_ [°C]	ΔH_mPCL_ [J/g]	T_ccPLA_ [°C]	ΔH_ccPLA_ [J/g]	T_mPLA_ [°C]	ΔH_mPLA_ [J/g]
PLA	297.2	56.4	-	-	-	-	126.6	11.2	150.5	11.4
PLA/PCL 85/15	318.9	55.8	41.5	1.3	56.2	3.7	123.9	14.5	149.6	15.8
PLA/PCL 85/15 1% TA	324.2	57.0	29.5	2.0	55.5	4.0	129.4	4.0	150.6	3.8
PLA/PCL 85/15 5% TA	315.3	56.6	23.9	9.3	55.0	7.5	128.3	7.1	150.1	6.3
PLA/PCL 70/30	311.7	56.2	35.1	14.0	56.9	14.0	125.7	8.0	149.7	8.3
PLA/PCL 70/30 1% TA	309.8	55.8	30.6	12.8	55.9	12.2	127.6	7.5	149.7	7.2
PLA/PCL 70/30 5% TA	315.9	54.8	25.0	10.1	52.8	10.0	128.4	5.7	150.1	6.3

**Table 5:** The reductions of the numbers of living and viable cells of *Escherichia coli*.

Sample	W	R	Biocidal Effect
PLA	-	0	no
PLA/PCL 85/15	5.0 × 10^5^	0	no
PLA/PCL 85/15 1% TA	9.8 × 10^4^	1	no
PLA/PC 85/15 5% TA	2.8 × 10^3^	2	yes
PLA/PCL 70/30	2.0 × 10^5^	0	no
PLA/PCL 70/30 1% TA	5.0 ×10^5^	0	no
PLA/PCL 70/30 5% TA	<10 × 10^0^	5	yes

W—Number of bacterial cells after contact time; R—reduction degree.

**Table 6:** The reductions of the numbers of living and viable cells of *Staphylococcus aureus*.

Sample	W	R	Biocidal Effect
PLA	-	0	no
PLA/PCL 85/15	1.5 × 10^6^	0	no
PLA/PCL 85/15 1% TA	1.8 ×10^5^	1	no
PLA/PC 85/15 5% TA	1.5 × 10^5^	1	no
PLA/PCL 70/30	1.5 × 10^6^	0	no
PLA/PCL70/30 1% TA	4.8 × 10^3^	3	yes
PLA/PCL 70/30 5% TA	1.3 × 10^1^	5	yes

W—Number of bacterial cells after contact time; R—reduction degree.

## Data Availability

Dataset available on request from the authors.

## Supplementary Materials

The following supporting information can be downloaded at https://www.mdpi.com/article/10.3390/polym17091134/s1, Table S1. Thermogravimetric analysis results.
