# Supplementary material for: PLA/PCL Polymer Material for Food Packaging with Enhanced Antibacterial Properties
Source: Polymers (Basel). 2025 Apr 22;17(9):1134. doi: 10.3390/polym17091134 (PMC12073487; doi:10.3390/polym17091134)
Supplement: Supplementary file 1 [file polymers-17-01134-s001.zip › polymers-3591688-supplementary.pdf]

Table S1. Thermogravimetric analysis results

**Sample**	**T_d [°C]**	**$T_{10\%}$ [°C]**	**$T_{50\%}$ [°C]**	**$T_{95\%}$ [°C]**	**R [%]**
PLA	297.2	308.5	332.7	349.0	0.0
PLA/PCL 85/15	318.9	327.0	347.5	371.9	0.0
PLA/PCL 85/15 1% TA	324.2	332.2	354.2	391.0	0.0
PLA/PCL 85/15 5% TA	315.3	325.7	349.8	403.3	0.1
PLA/PCL 70/30	311.7	321.7	345.7	394.4	0.4
PLA/PCL 70/30 1% TA	309.8	318.5	343.8	395.4	0.6
PLA/PCL 70/30 5% TA	315.9	328.5	357.3	412.8	0.0
